# On the MLC leaves alignment in the direction orthogonal to movement

**DOI:** 10.1002/acm2.13267

**Published:** 2021-05-30

**Authors:** Kujtim Latifi, Rajiv Lotey, Vladimir Feygelman

**Affiliations:** ^1^ Department of Radiation Oncology Moffitt Cancer Center Tampa FL USA; ^2^ ViewRay Inc. Mountain View CA USA

**Keywords:** IMRT/VMAT verification, MLC acceptance testing, MLC modeling, MLC QA

## Abstract

The main focus of the recommended spatial accuracy tests for the multi‐leaf collimators (MLC) is calibration of the leaf position along the movement direction and overall alignment to the radiation isocenter. No explicit attention was typically paid to the alignment of the leaves from the opposing banks in the direction orthogonal to movement. This paper is a case study demonstrating that verification of such alignment at the time of acceptance testing is prudent. The original standard MLC (SMLC) on an MRIdian MRI‐guided linac (ViewRay Inc., Mountain View, CA, USA) was upgraded to a high‐speed MLC (HSMLC), which is supposed to be mechanically identical to the SMLC except for the higher drive screw pitch. The results of the end‐to‐end IMRT tests demonstrated unacceptable dosimetric results exemplified by an average and maximum ion chamber (IC) point dose error in the high‐dose low‐gradient region of 2.5 ± 1.4% and 4.6%, respectively. Before the upgrade, those values were 0.3 ± 0.7% and 0.9%, respectively. An exhaustive analysis of possible failure modes eventually zeroed in on the average misalignment of about 1 mm in the Y (along the couch) direction between the right and left upper MLC banks. The MLC was replaced, reducing the Y‐direction misalignment to 0.4 mm. As a result, the average and maximum IC dose‐errors became acceptable 1.0 ± 0.7% and 1.6%, respectively. Simple film and/or chamber array tests during acceptance testing can easily detect Y‐direction misalignments between opposing leaves banks measuring a fraction of a mm at isocenter. Left undetected, such misalignment can cause nontrivial dosimetric consequences.

## 1 INTRODUCTION

The suggested acceptance and periodic quality assurance procedures for the multi‐leaf collimators (MLC) have been distilled to the list of practical approaches and tests in the professional guidance documents.^1^, ^2^ The main focus of the spatial accuracy tests is calibration of the leaf position along the movement direction and alignment of the MLC to the radiation isocenter. An elaborate test for the latter was suggested by Losasso^3^ but the collimator “spoke shots” are employed more commonly. To the best of our knowledge, the possible misalignment in the direction orthogonal to movement between the leaves from the opposing MLC banks has not been explicitly addressed before. This paper is a case study demonstrating that during acceptance testing this alignment should be verified.

The MRIdian MRI‐guided linac radiotherapy system (ViewRay Inc, Mountain View, CA, USA) has been in service at our institution for over a year. It underwent rigorous dosimetric commissioning including a successful independent end‐to‐end audit with an MRI‐visible head and neck mail‐in dosimetry phantom from IROC Houston.^4^ This work was precipitated by a scheduled upgrade of the original standard MLC (SMLC) to a high‐speed one (HSMLC). The new design is virtually identical to the SMLC, except the pitch of the drive screws is approximately four times higher, resulting in a proportional increase in the linear leaf speed. No change to the dosimetric characteristics of the MLC beyond normal manufacturing tolerances is implied in the design. Two HSMLCs were studied in this work in comparison with the original SMLC. High‐speed MLC#1 demonstrated unintended behavior that prompted the current investigation and was ultimately replaced with HSMLC#2, currently commissioned for clinical use.

## 2 METHODS

### 2.1 The MLC

**Fig. 1:**
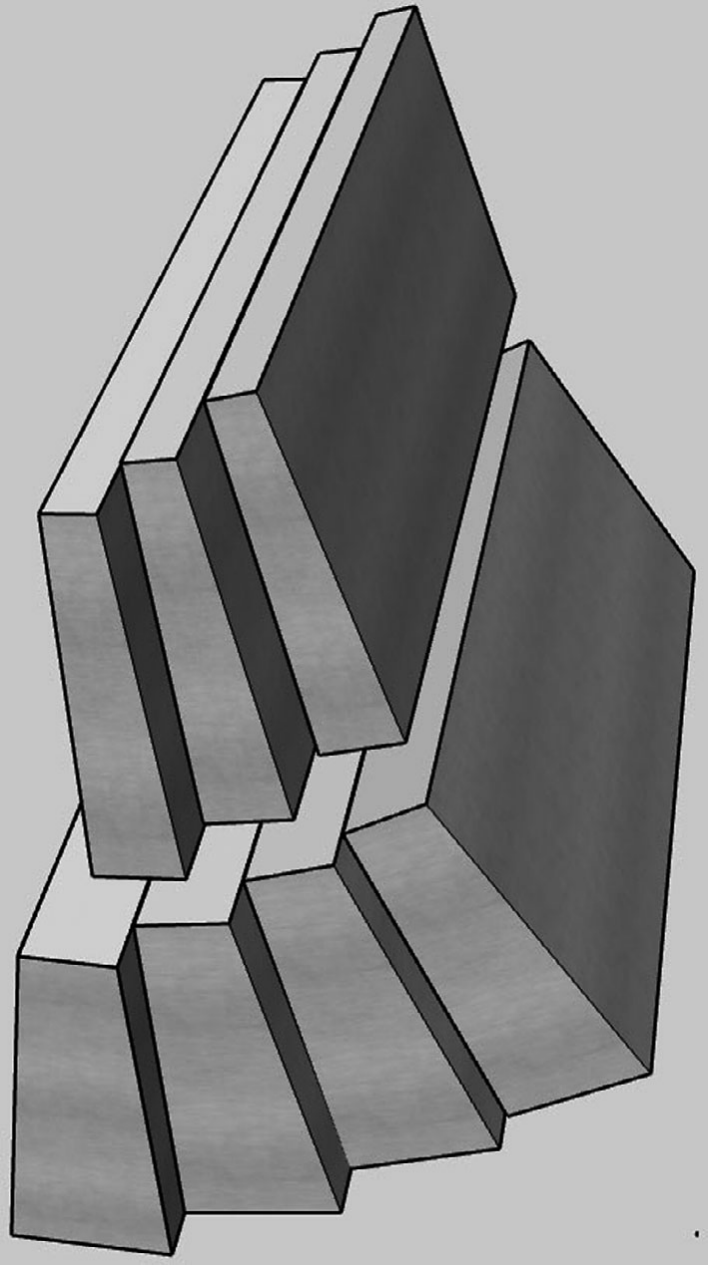
A schematic representation of a portion of the dual‐layer staggered MRIdian linac MLC.

The MLC design on a single‐head linac‐based system is different from the three‐head ^60^Co unit.^5^ The defining feature is the double‐stack double‐focused configuration with an individual leaf width of 8.3 mm as projected at the isocenter (Fig. 1). The leaf sides are flat, without a tongue‐and‐groove arrangement. The leaves are chamfered at the tip to facilitate smoother interdigitation. They can travel across the entire field width. The MLC is the only variable beam‐shaping device in MRIdian. There is no field light and the MLC does not rotate.

### 2.2 MLC tests

The MLC tests can be divided into two groups by the timeline: the standard tests included in the acceptance test of the new MLC and the additional tests introduced after the initial failure of the IMRT dosimetric evaluation with HSMLC#1.

The standard tests included picket fence films at three cardinal gantry angles for each MLC layer, combined MLC transmission and intraleaf leakage measurements with a Farmer chamber at isocenter for each layer, and a radiochromic film leakage measurement with both layers closed, to locate any potential unintended hot spots. Also included in this group of MLC‐related checks were the gantry star shot with radiochromic film and a complementary gantry isocentricity test with an ion chamber array.^6^ The latter two are used to define the radiation isocenter position at the time of installation.

**Fig. 2:**
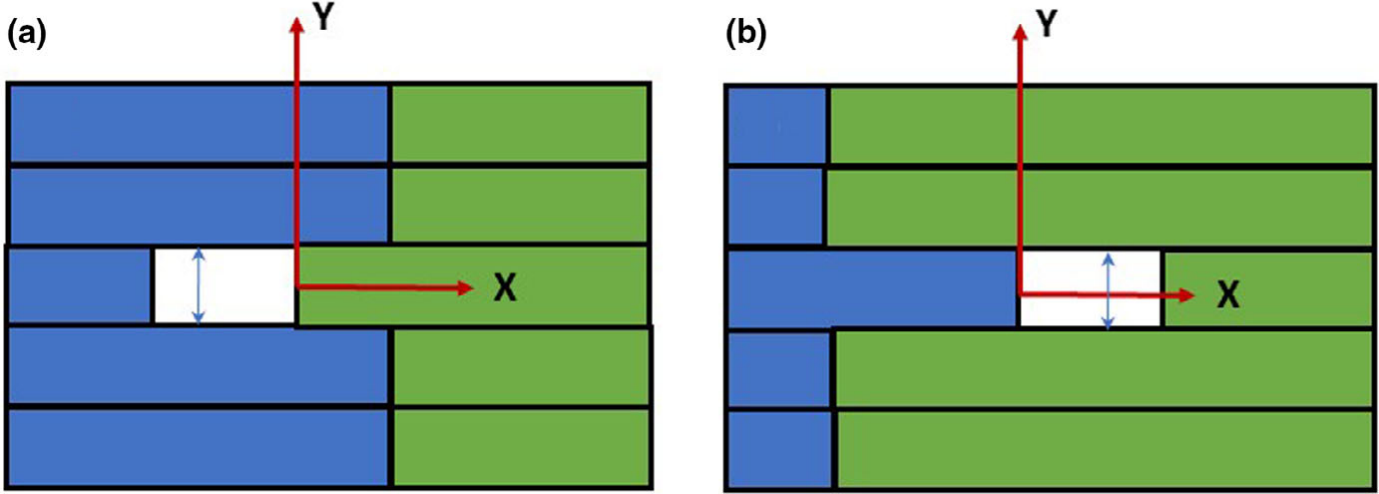
Abutting openings with the Y edges defined by the leaves from the left (a) or right (b) banks, shown in relation to the isocenter. The left bank leaves are depicted in blue and the right bank in green.

**Fig. 3:**
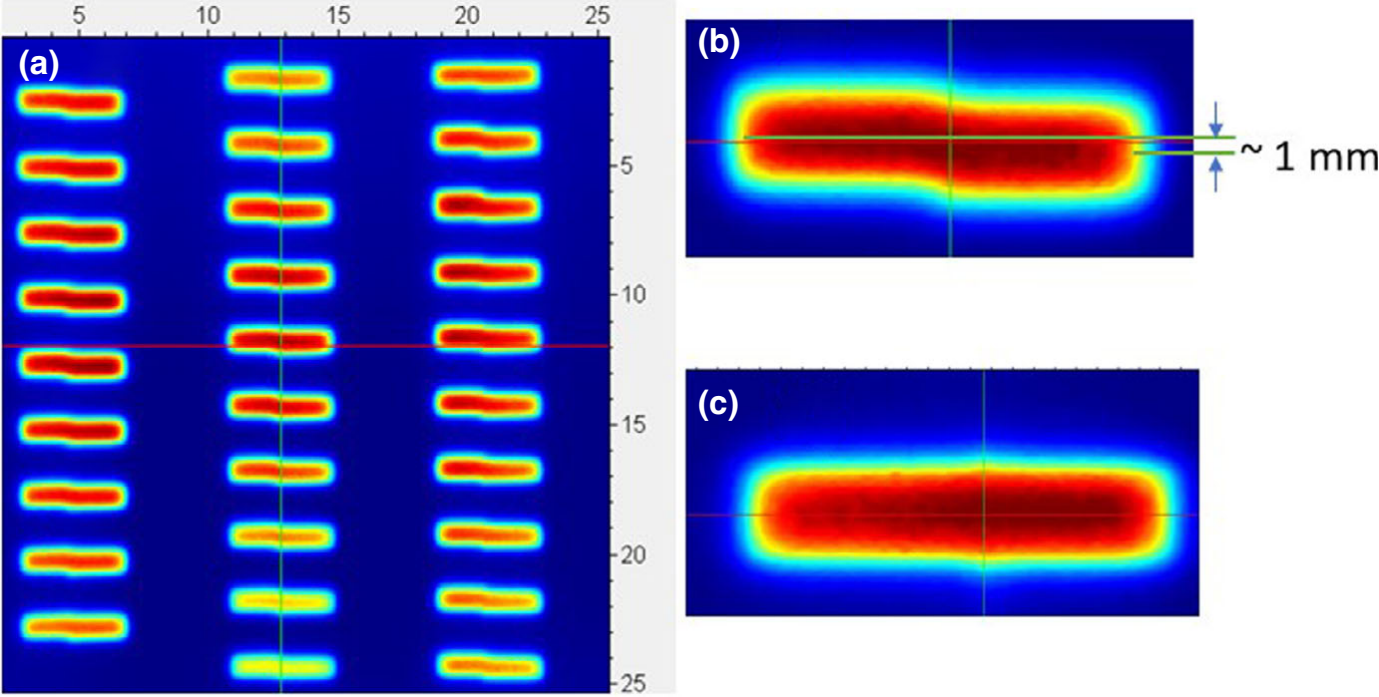
Relative dose on the calibrated radiochromic film exposed to multiple abutting fields defined by the left and right leaf banks of the upper MLC layer of HSMLC#1. (a): An overview; (b): A central axis blow‐up demonstrating a shift between the left and right leaf banks Y position in HSMLC#1. (c) The same as (b) but with better aligned HSMLC#2.

As an additional test, a simple MLC check was designed to show any potential misalignment between the leaves in the left and right banks in the Y‐direction (IEC Standard 61217 Y, along the couch). For each MLC layer, the pairs of abutting openings were created. Each one was one leaf width tall (8.3 mm) and 20 mm wide. The superior/inferior borders of the fields to the left of the abutment line were defined by the left leaf bank while the right bank was used for the other field (Fig. 2). Multiple opening pairs were constructed throughout the collimator opening to test a range of leaf pairs and lateral positions. (Fig. 3). A sheet of radiochromic EBT3 film (Ashland Advanced Materials, Bridgewater, NJ, USA) was exposed at 1.5 cm depth, scanned on a flatbed scanner at 150 dpi, calibrated and analyzed in RIT113 v. 6 software (Radiological Imaging Technologies, Colorado Springs, CO, USA). The dosimetric center of the opening in the Y direction was determined from the dose profiles through the centers of the left and right abutting fields.

A complementary test was conducted with the MR‐compatible IC Profiler (ICP‐MR, Sun Nuclear Corp, Melbourne, FL, USA). With the device aligned on the lasers, the half‐beam split fields (27.4 cm^2^ × 12.035 cm^2^) were created, again with either the left or the right central leaf defining the Y edge of the field. The profiles along the X direction were recorded at 1.5 cm depth, representing the dose ratio between the two apertures. If both left and right leaves’ lateral edges projected at the same Y position, the signals would be equal. A shift between the banks would result in the different volumes of the 4 mm‐long chambers being irradiated to high dose, and hence in signal differences. To quantify the effect, the couch was translated in the Y direction in 0.2 mm increments under the half‐beam opening produced by HSMLC#2.

The relative signal was plotted against the table shift and the slope of the linear fit indicated the signal gradient in the Y direction. The signal was defined as an average of the readings of the two chambers ±1 cm from the central axis. Their long axes were oriented in the Y direction. The MRIdian table position readout resolution is 0.1 mm. It has been shown previously that the Y‐position readout correlated tightly with the radiation beam center measurement.^6^

### 2.3 Dosimetric IMRT tests

MRIdian allows only static gantry step‐and‐shoot IMRT. The test plans were developed at the initial commissioning of the system and consisted of the 4‐plan test suite from TG119 ^7^ and three cases from MPPG 5a^8^ – head and neck, prostate bed, and abdomen. The average number of beams was 13 (range 7–19) delivering an average of 120 segments (80–145). The average monitor units (MUs) per 1 cGy of target dose was 6.1 (4.1–9.1). The plans were deliberately allowed to have a substantial number of segments small in size and/or duration (2 MU/segment minimum).

Point dose (ion chamber) measurements were performed in a 20 × 20 × 20 cm^3^ Plastic Water Cube phantom (CIRS Inc, Norfolk, VA, USA). A Model 31010 Semiflex 0.125 cc ion chamber (PTW, Freiburg, Germany) was kept at the isocenter except for the TG119 multi‐target cylinder plan where it was also shifted ±4.5 cm in the Y direction. The chamber daily correction factors were obtained by cross‐calibration to the ViewRay TPS dose at isocenter in the phantom in the parallel‐opposed 10 × 10 cm^2^ fields.

Dose‐distribution measurements were performed with a helical diode array – the ArcCHECK‐MR (AC‐MR, Sun Nuclear)^9^ with SNC patient software v. 8.3. The dosimeter was cross‐calibrated daily in the parallel opposed fields to minimize differences in the central portion of a corresponding plan. The results of the gamma analysis comparison were reported with the standard 3% dose‐error with global normalization /2 mm distance to agreement criteria (3%G/2mm),^10^ as well as with 2% local (L) dose‐error threshold.

The differences in median values were tested for statistical significance using the GraphPad Prism software v. 8 (GraphPad Software, San Diego, CA). When three sets of data were available (IC measurements), a nonparametric version of the ANOVA test was used (Friedman’s test) followed by multiple comparisons to the control column (Dunn’s test).^11^ For the two datasets comparison (AC‐MR) Wilcoxon’s signed rank test was employed.^11^ P‐values ≤0.05 indicated statistical significance.

## 3 RESULTS

### 3.1 MLC tests

Starting with the standard acceptance tests, the HSMLC#1 picket fence films demonstrated acceptable results at 0, 90, and 270⁰ gantry angles. The absolute positions of the leaf edges (50% of the dose profile) with respect to the isocenter laser line did not deviate from the nominal values by more than 0.45 mm.

The transmission coefficients at the central axis for individual MLC layers are presented in Table 1 for the SMLC and two replacement HSMLCs. Some differences were expected, as the dosimetric interleaf spacing depends on a number of mechanical parameters and varies between different MLCs of the same design and between different leaf pairs of the same MLC. The difference between the upper and lower layers is due to the different upper and lower interleaf gap patterns intercepted by the horizontally positioned chamber with an active volume length of ~20 mm. The upper leaves straddle the central axis while the lower leaves are centered on it. The leakage film with both MLC layers closed did not reveal any unexpected hot spots. The radiation field center position in the Y direction, as measured by the ICP‐MR irradiated from four cardinal gantry angles,^6^ varied by just 0.1 mm. A film‐based gantry star shot demonstrated that the radiation field center was confined to a 0.43 mm radius circle in the transverse plane, comparing favorably to the specification of 1.0 mm. Overall, the standard tests of HSMLC#1 did not indicate any potential problems.

**Table 1:** Transmission coefficients (an average of the right and left banks) on the central axis for individual MLC layers, measured with a Farmer chamber.

	Original	HSMLC#1	HSMLC#2
Upper	0.054	0.050	0.054
Lower	0.033	0.035	0.036

**Fig. 4:**
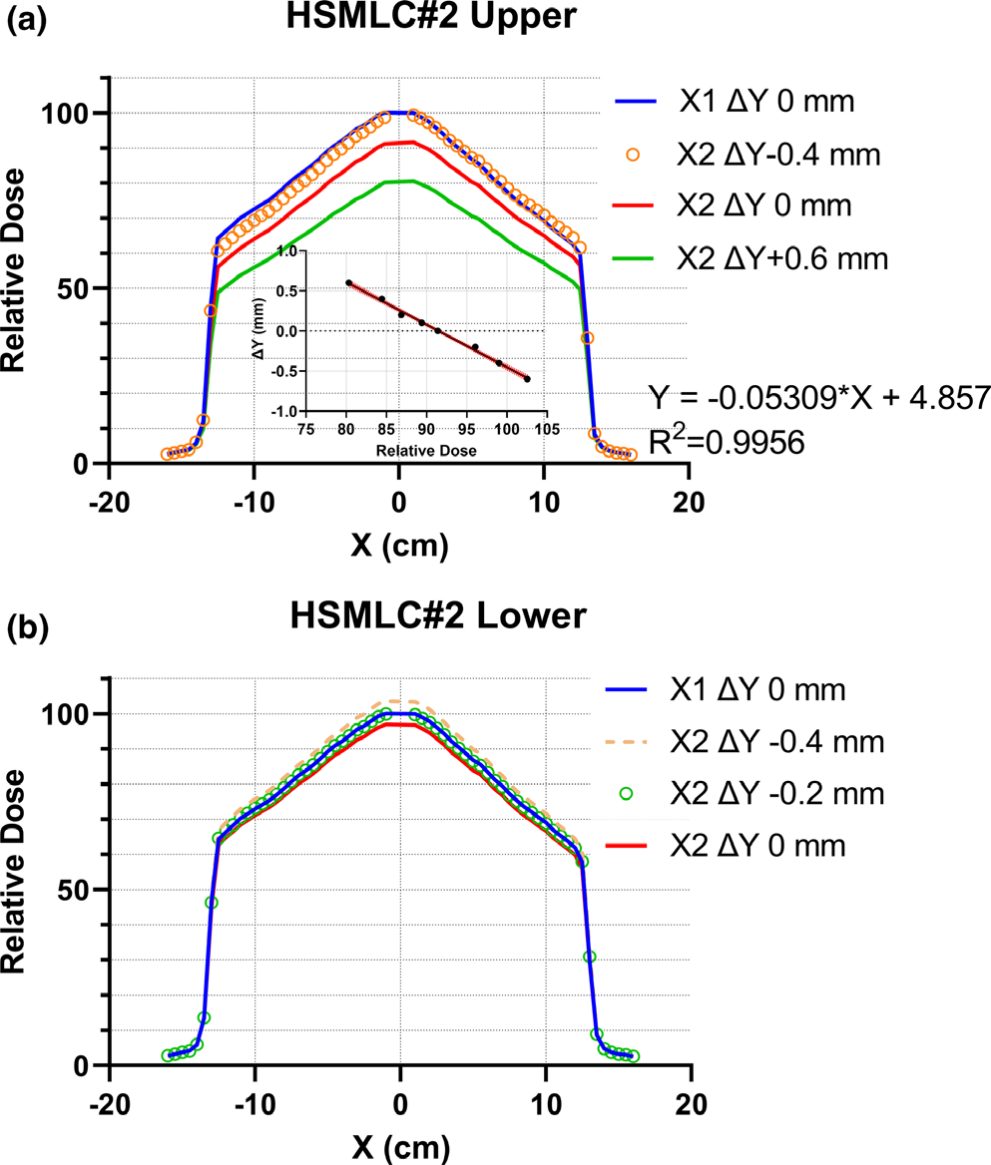
The X‐axis profiles through the central axis with the half‐beam border defined by a leaf from the X1 (left) vs. X2 upper MLC bank. (a): Upper MLC layer; Insert: linear regression of the table shift ΔY vs. central chambers average signal, with the 95% confidence band. ΔY = 0 corresponds to the cross‐hairs in the center of the array aligned with the lasers at the isocenter. (b): Lower MLC layer.

However, after the extensive investigation of the possible causes of the unsatisfactory end‐to‐end IMRT test results, additional tests beyond the standard suite were performed. The abutting fields defined by either left or right leaves of the upper MLC layer revealed an average 1 mm shift in the Y direction between the left and right (X1 and X2) banks [Fig. 3(a)]. In the center of the MLC the shift was 1.3 mm [Fig. 3(B)]. A similar film with the lower layer of HSMLC#1 failed to demonstrate a comparable shift. After HSMLC#2 was installed, the Y‐positioning film demonstrated a substantially reduced shift, estimated to be 0.4 mm at the central axis. Since HSMLC#2 was permanently mounted, it was possible to analyze it in greater detail. A subset of profiles for the upper MLC layer obtained with the different table shifts in the Y direction is presented in Fig. 4(a). The signal for the X1‐defined beam edge and the initial table position was arbitrarily assigned a value of 100%, while the table was shifted when the half‐beam border was defined by the X2 leaf. A shift of 0.45 mm equalized the signals, comparing favorably to the 0.4 mm shift value obtained from the film measurements. The insert on Fig. 4(a). demonstrates that around the center of a 4 mm long ion chamber a signal gradient of the order of 19%/mm can be expected. This has clear implications for IMRT dosimetry. The lower MLC layer demonstrated a borderline‐detectable shift between the X1 and X2 banks of 0.2 mm [Fig. 4(b)].

The precision of the ICP measurements can be estimated from the goodness‐of‐fit parameters for the ΔY vs. relative dose regression line in the insert on Fig. 4(a). The correlation coefficient R^2^ = 0.9956. There is no observed statistically significant deviation from linearity (runs test *p* = 0.43). The 95% confidence interval of ΔY when ΔY = 0 is ±0.05 mm and the standard error of the ΔY residuals is 0.03 mm. Such precision is sufficient to estimate shifts of >0.2 mm described in this paper. The upper layer shift measured with film (0.4 mm) is within the 95% confidence interval of the ICP measurement (0.45 mm).

### 3.2 Dosimetric IMRT Tests

The ion chamber measurement results for three MLCs are summarized in the first part of Table 2. The Friedman’s test revealed overall statistically significant differences between IC readings with the SMLC, HSMLC#1, and HSMLC#2 (*p* = 0.0007). The follow‐up multiple comparisons to the control set (SMLC) yielded statistically significant difference for HSMLC#1 (*p* = 0.0008) but insignificant one for HSMLC#2 (*p* = 0.31). The difference in the average passing rate is minimal (0.5%) for the accepted clinical 3%G/2mm criteria combination.^10^ It increases to 2.4% for the more sensitive 2%L/2 mm criteria. However even with these criteria the AC‐MR gamma analysis passing rates were not statistically different between the SMLC and HSMLC#2 (*p* = 0.09).

**Table 2:** Detailed results of the ion chamber and AC‐MR dose comparisons with the TPS for IMRT test plans delivered with different MLCs. The gamma analysis was performed with 3% Global (G) and 2% Local (L) dose‐error normalization.

	IC Dose‐difference (measured‐calculated), %			AC‐MR γ‐analysis pass rate (%)			
				SMLC		HSMLC#2	
	SMLC	HSMLC#1	HSMLC#2	3%G/2mm	2%L/2mm	3%G/2mm	2%L/2mm
TG119 Prostate	−0.7	−4.6	−1.6	95.0	86.9	95.9	85.9
TG 119 C Shape	−0.5	−2.4	−0.8	96.6	90.3	95.8	87.3
TG119 HN	−1.0	−1.7	−1.1	95.8	87.4	98.6	89.5
TG119 MTgt 100%	0.9	−1.5	−0.5	99.5	92.2	98.9	87.1
TG119 MTgt 50%	−2.0	−1.7	−0.6	−	−	−	−
TG119 MTgt 25%	1.2	−4.9	−2.4	−	−	−	−
TG244 Abd	−0.4	−3.2	−0.8	98.7	85.8	98.9	85.8
TG 244 Prost Bed	0.5	−0.6	0.0	99.2	94.2	99.9	86.9
TG244 HN	−0.3	−2.3	−1.1	100.0	95.8	100.0	93.2
Ave	−0.3	−2.5	−1.0	97.8	90.4	98.3	88.0
SD	1.0	1.4	0.7	2.0	4.1	1.9	2.8

## 4 DISCUSSION

The average ion chamber IMRT dose difference of −2.5% with the replacement HSMLC#1 was in contrast to the original value of −0.3%. An average disagreement between the calculated and measured dose in excess of 2% is considered unacceptable, while the desirable value is below 1.5%.^8^ The cause of the excessive disagreement was investigated and a number of potential standard culprits were sequentially eliminated, including test instrumentation faults, gradients across the PTW ion chamber, monitor unit non‐linearity, beam energy variation, output and beam shape variation with gantry angle, isocenter wobble, the MLC X‐direction leaf position variation with gantry angle, and change in MLC leakage. Finally, it was noticed that the X1 and X2 (left and right) banks of the upper layer in HSMLC#1 were misaligned in the Y direction by 1 mm on average (Fig. 3). That immediately resulted in a reasonable explanation of the unusually large error observed with the TG119 Mock Prostate test plan (−4.6%). With the chamber positioned at the isocenter, that plan contained the largest number of segments with the upper MLC leaves splitting the detector active volume (6.5 mm long) approximately in half. Given the large dose‐difference when either the X1 or the X2 leaves were splitting the chamber, a small imbalance in the number of MUs delivered in each configuration could easily lead to a few percent error in the final dose when the leaves are misaligned by ~1 mm. The ViewRay treatment planning system (TPS) assumes that the Y position of any leaf equals the nominal. Thus, there are no adjustable parameters in the TPS to correct for the substantial misalignment and the only remedy was to exchange the MLC. The replacement HSMLC#2 had substantially smaller Y‐direction misalignment of the opposing upper leaves (0.4 mm vs. 1 mm) and produced satisfactory dosimetric results (Table 2), largely in line with the original SMLC.

Another parameter potentially affected by the Y‐direction leaf misalignment is the measured radiation isocenter position. With MRIdian, it is typically established with the movable leaves defining the upper and lower Y borders of the radiation field. Depending on which layer and which leaf bank happen to define the field of a particular size, different values can result, increasing the uncertainty of the radiation isocenter location.

The standard acceptance tests at the time were not well geared towards detecting a potential Y‐direction misalignment between the leaves from the opposing banks. Such misalignment can be detected easily with film or ion chamber measurements described in this report. If a site has access to an ICP, the slope value from Fig. 4 can be used to directly convert the signal difference into the geometrical distance between the edges of the left and right bank leaves. If using another ion chamber, the slope has to be determined first as it depends on the detector size.

The vendor failure mode analysis determined the cause of the misalignment to be a shift in one of the mechanical tension guides confining the leaf bank Y position. The damage has likely occurred during shipping, as the shock sensor had been tripped. The MLC undergoes rigorous testing at the factory and such a misalignment would be unlikely to pass the inspection.

## 5 CONCLUSIONS

The alignment between the opposing leaf banks in the direction orthogonal to movement has not received much attention in the literature, perhaps because of the tacit assumption that in the absence of movement the mechanical specifications of the MLC assembly were sufficiently tight. However, with a certain MLC design such misalignment can be systematic across the radiation field and lead to measurable dosimetric consequences. Simple film and/or chamber array tests at the time of acceptance can easily detect the Y‐direction misalignments between opposing leaves measuring a fraction of a mm at isocenter.

## Conflict of interest

Rajiv Lotey is an employee of ViewRay Inc. Moffitt Cancer Center has a Research Agreement with ViewRay Inc. No funding was received for this work and there is no specific potential conflict of interest to report.

## Author Contribution

K. Latifi — experimental design, execution, and manuscript approval. R. Lotey — experimental design, execution, and manuscript approval. V. Feygelman — experimental design, execution, and manuscript drafting and approval.

## Data Availability

Data available upon reasonable request from the authors.
